# Betalain Pigments: Isolation and Application as Reagents for Colorimetric Methods and Biosensors

**DOI:** 10.3390/bios15060349

**Published:** 2025-06-01

**Authors:** Rimadani Pratiwi, Devita Salsa Maharani, Sarah Gustia Redjeki

**Affiliations:** Department of Pharmaceutical Analysis and Medicinal Chemistry, Faculty of Pharmacy, Universitas Padjadjaran, Sumedang 45363, Indonesia; devita21001@mail.unpad.ac.id (D.S.M.); sarah21006@mail.unpad.ac.id (S.G.R.)

**Keywords:** betalains, betacyanins, betaxanthins, isolation, colorimetric sensor, biosensor

## Abstract

Betalains are hydrophilic natural pigments commonly found in plants of the *Caryophyllales* order, as well as in specific species and genera of fungi, such as *Hygrocybe*, *Hygrophorus*, and *Amanita muscaria*. Betalains are sorted into two groups: betacyanins, which form red-violet pigments, and betaxanthins, which form yellow-orange pigments. These compounds can be employed as colorimetric sensors and biosensors. This paper provides a review of the isolation methods of betalains and the various applications of betalains as colorimetric sensors and biosensors. The review was conducted by collecting publications over the last decade. The results show that betalains can be used as a colorimetric sensor to identify metal compounds in water and nonmetal compounds that indicate the quality of food. In addition, betaxanthin has been used for developing cell-based biosensors from yeast and bacteria. Furthermore, betalain as a colorimetric sensor and biosensor is developed by using an innovative digital detector, such as a smartphone. Nevertheless, the fragile stability of betalains presents a significant barrier during the extraction. As a result, future studies could focus on adding innovative technologies for optimizing extraction and also developing betalain as novel bio-indicators for specific analytes.

## 1. Introduction

Analytical chemistry methods are used broadly in academic and applied laboratories. Analytical chemistry as a science aims to develop techniques that provide the necessary information about a sample with analytical accuracy, precision, sensitivity, and selectivity. On the other hand, solvents, reagents, and energy are required in performing said analytical methods, and they generate waste [1,2]. Recently, the development of environmentally friendly conscientious analytical chemistry has emerged. One of the principles of green analytical chemistry is safer chemicals, which implies eliminating or replacing toxic or hazardous solvents and reagents [2]. This can be accomplished by employing natural pigments as reagents. Natural pigments are naturally occurring colorants that can be obtained from plants, animal matrices, or microorganisms. According to their structure, natural pigments are classifiable into various groups, including compounds with distinct features, for example, derivatives of isoprenoids (iridoids and carotenoids), quinones (benzoquinone, naphthoquinone, and anthraquinone), benzopyran derivatives (anthocyanins and other flavonoids), derivatives of tetrapyrrole (chlorophylls and heme colors), *N*-heterocyclic compounds dissimilar from tetrapyrroles (betalains, flavins, purines, pterins, phenazines, and phenoxazines), and melanins [3,4]. Among them, the most fundamental are either hydrophilic or lipophilic compounds, exemplified by carotenoids, betalains, chlorophylls, and anthocyanins [4].

Betalains are water-soluble natural pigments frequently found in beetroot (*Beta vulgaris*), cactus pear (*Opuntia* spp.), djulis (*Chenopodium formosanum*), amaranth (*Amaranthaceae*), pitayas (*Stenocereus* ssp.), and pitahayas (*Hylocereus undatus*) [5]. Betalains can also be found in bracts, stems, and flowers of edible plants to be utilized as natural colorants. Plants abundant in betalains exhibit high anti-inflammatory as well as antiproliferative properties and possess health-promoting benefits in humans. Betalain pigments are categorized into two groups: betacyanins, which form red-violet pigments, and betaxanthins, which form yellow-orange pigments [6,7]. Betalain molecules carry two nitrogen atoms in their basic structure. Betalamic acid is the chromophore of all betalain molecules. Betacyanin aglycones are arranged from cyclodihydrophenylalanine (DOPA) or 2-descarboxy-cyclo-DOPA and betalamic acid. Betacyanins exhibit maximum light absorption within the visible spectrum (534–555 nm, depending on the solvent) [8]. The formation of betaxanthins results from the excess of some amines in the condensation of betalain. This process stimulated the reduction in betalamic acid amount in solution, which suggested that there is an increase in betaxanthin stability [7]. Betaxanthins are fluorescent molecules, characterized by excitation maximum in the 463–535 nm range and a peak emission within the 508–608 nm range [9].

**Figure 1:**
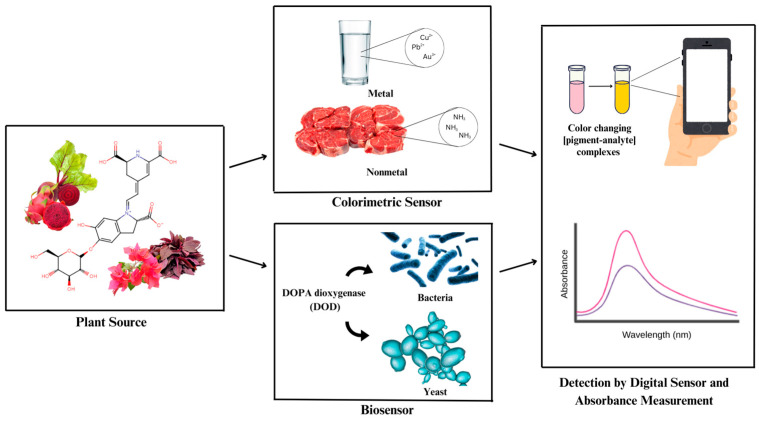
General scheme of betalain utilization as a colorimetric sensor and biosensor.

Up to this point, there has just been a single review that covers the chemical properties and uses of betacyanin [10]. In this review, we present an overview of the isolation methods as well as the various applications of betalains as colorimetric sensors and biosensors (Figure 1). Recent advancements in betalain isolation methods, ranging from traditional solid–liquid extraction to modern techniques like microwave- and ultrasound-assisted extraction, and supercritical fluid extraction, have improved yield and stability, thereby enhancing their suitability for analytical applications. Colorimetric sensors are a type of optical sensor that changes color when impacted by external factors [11]. The stability of betalain as colorimetric sensors is significantly decreased by a number of critical factors, including pH, exposure to light, oxygen, or high temperatures [12]. Due to their advantages in quickness and ease of preparation without requiring high-tech instruments, wide dynamic range, fast reaction, high sensitivity and selectivity at optimal conditions, and affordability, colorimetric sensors have caused significant concerns [13]. Colorimetric sensors have been applied to the detection of biomolecules, organic pollutants, heavy metal ions, and chemicals [14]. Moreover, biosensors are tools for analysis that convert biological reactions into comprehensible electrical signals. Biosensors have made use of biological reactions such as the production of specific reporter proteins, changes in the amount of carotenoids, fluorescence from photosynthetic pigments, and enzyme activity [15]. Recent studies have demonstrated the use of genetically engineered microbial systems to produce visible and quantifiable betalain fluorescence in response to specific analytes such as copper ions, amino acids, and other chemical substances with good performance [16,17,18,19,20,21,22].

## 2. Characterization of Betalain

### 2.1. Source of Betalain

Betalains are pigments found in about 17 plant families under the *Caryophyllales* order. The families *Amaranthaceae*, *Basellaceae*, *Cactaceae*, *Portulacaceae*, and *Nyctaceae* belong to the *Caryophyllales* order. Certain fungus species and genera, including *Hygrocybe*, *Hygrophorus*, and *Amanita muscaria*, are also known to have these pigments [23]. In the *Caryophyllales* family, betalains are most commonly found in edible sources in red beet roots (*Beta vulgaris* L.). Nitrate, vitamins, minerals, phenolics, carotenoids, and ascorbic acid are all abundant in this crop [24]. It was frequently believed that red beet roots (*Beta vulgaris* L.) are a unique source of betalains because they contain the two major betalain pigments, red betanin and yellow vulgaxanthin I [25].

Other plant sources used to produce betalain include pitaya fruits (*Hylocereus andatus*), some species of grainy or leafy amaranth (*Amaranthus* sp.), fruits of cacti *Opuntia* sp., *Eulychnia* sp., and *Hylocereus* sp., including the dragon fruits of primarily *Hylocereus polyrhizus* (Web.) Britton and Rose and related species, and colored Swiss chard (*B. vulgaris* L. ssp. cicla). A few less common sources are the bloodberries (berries of *Rivina humilis* L.), *Phytolacca americana* L., *Talinum triangulare* (Jacq.) Willd., *Amaranthus cruentus* L., *A. coudatus* L., and *A. hybridus* L., and the tubers of ulluco (*Ullucus tuberosus Caldas*) [25,26].

### 2.2. Classification of Betalain

**Figure 2:**
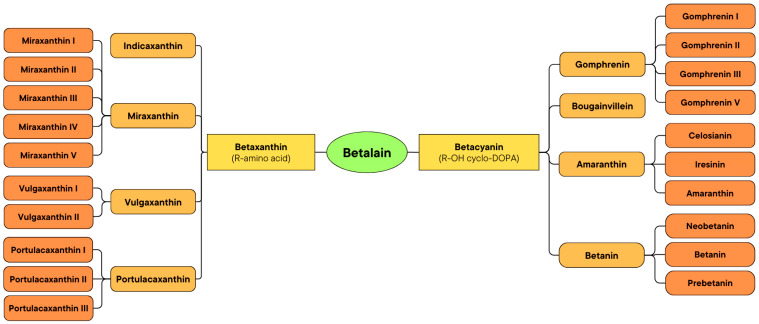
Scheme for classification of betalains.

Betalains are classified into two subclasses, betacyanins and betaxanthins, which are illustrated in the schematic diagram in Figure 2, while their respective chemical structures are shown in Table S1 in the Supplementary Information. The reddish-violet betalain pigments known as betacyanins have an absorbance range of 535–550 nm [25]. Plants store them in vacuoles as glycosides. Betacyanins are the result of the condensation of betalamic acid and cyclo-DOPA. However, because light-sensitive enzymes are used in their manufacturing process, they are sensitive to both light and temperature. Several types of betacyanin are divided into the betanin group, amaranthin group, gomphrenin group, and bougainvillein group. Betanin is the most prevalent kind of betacyanin. Large amounts of it can be found in beets and pokeberry. It exists as a glucoside that, upon hydrolysis, transforms into a sugar unit and betanidin [27].

Betaxanthins, also known as flavocyanins, are yellow to orange pigments that have a maximum absorbance at 482 nm [28,29,30,31]. Betaxanthins are produced when betalamic acid reacts with an amino acid or amine. Due to the sensitivity of the enzymes required for biosynthesis to light and temperature, both of these have an effect on their synthesis. Betaxanthins are kept in the vacuoles of amaranthus plants after synthesis. The ability to autofluoresce allows betaxanthins to be distinguished from betacyanins. Types of betacyanin are divided into indicaxanthin, vulgaxanthin, and portulacaxanthins [27].

### 2.3. Isolation of Betalain

**Figure 3:**
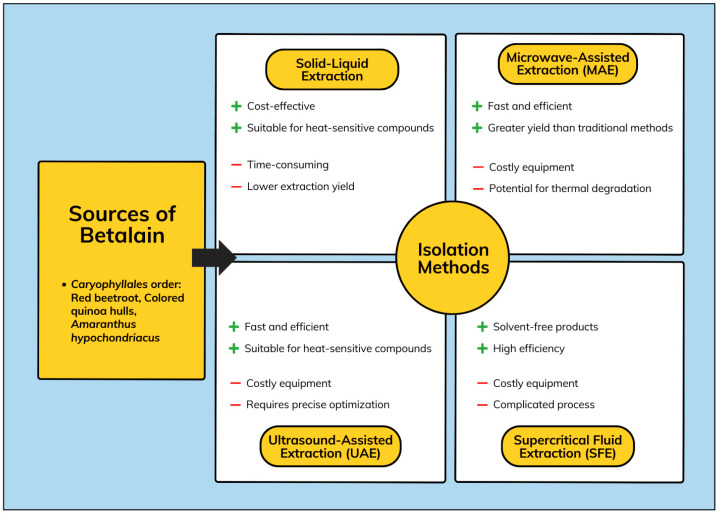
General scheme for the isolation methods of betalain.

Red beetroot (*B. vulgaris*) has long been considered a unique source of betalains. Betalain pigments can only be found in plants of the Caryophyllales family, except for Caryophyllaceae and Molluginaceae [32]. Due to their water-soluble nature, betalains can be extracted using pure water, ethanol, or aqueous methanol. Despite that, the concentration of individual constituents in the extract is affected by the extraction process as well as the selection of solvents. Additionally, optimizing the yield of betalains during isolation and processing is crucial, given that the isolated betalains are easily affected by pH, light, water activity, oxygen, temperature, enzymatic activities, and metal ions [33]. The general scheme for the isolation methods of betalains is shown in Figure 3. 

A variety of extraction techniques have been developed for the isolation of betalains, with each method offering distinct advantages and limitations. The traditional solid–liquid extraction method is simple, cost-effective, and suitable for heat-sensitive compounds, but it is time-consuming and has a lower extraction yield [34,35]. Microwave-assisted extraction (MAE) enhances extraction efficiency by providing higher yields within a shorter duration and requires less solvent [36]. However, it requires costly equipment and may pose a risk of thermal degradation to sensitive compounds [37]. Ultrasound-assisted extraction (UAE) also offers high efficiency and is suitable for heat-sensitive compounds, though it requires specialized equipment and careful optimization to ensure effective results [38,39,40]. Lastly, supercritical fluid extraction (SFE) offers solvent-free products and a highly efficient process, but is limited by the high cost of equipment and complicated process [41,42,43]. 

Betalains may be isolated from red beetroot by MAE, which is a promising substitute for traditional extraction. MAE is a commonly used method to isolate bioactive compounds from medicinal herbs by utilizing microwave energy to heat solvents that contain samples, causing the analytes to separate from the sample matrix and dissolve into the solvent [44]. MAE reduces solvent usage, processing time, and energy requirements, while simultaneously increasing yield. Cardoso-Ugarte et al. [36] performed the extraction of betalains from red beets using MAE, which involves combining 0.1 g of freeze-dried samples with 25 mL of an extraction solvent, consisting of a 1:1 ratio of ethanol to water, within 125 mL Teflon extraction vessels. The extraction conditions were set with chosen power levels (400, 800, and 1200 W), duty cycles (50, 100%), time (0–160 s), and agitation. Immediately following the extraction procedure, the red beet cubes were extracted from the solvent to avoid additional migration. If necessary, the volume was adjusted to 25 mL and directly cooled down in an ice water bath, preventing pigment decomposition due to temperature. After conducting the one-step MAE, it was found that longer microwave exposure increased the temperature, leading to thermal degradation of the extracted pigments. To maximize pigment extraction while minimizing betalain degradation and improving pigment preservation, a two-step MAE procedure was implemented, incorporating L-ascorbic acid into the extraction solvent. The two-step MAE process included a cooling phase in an ice water bath for five minutes following the first extraction, aiming to achieve a temperature of 23 ± 1 °C in the vessels. This cooling phase prevented the decomposition of the already obtained betalains and facilitated a subsequent MAE step for additional betalain extraction. This study showed that an integrated treatment of 400 W and 100% duty cycle (microwave is on for 100% of the time) for 90–120 s resulted in the greatest quantity of recovered betanins (128.68 mg per 100 g of freeze-dried red beet). In contrast, extraction times of 140 and 150 s resulted in the greatest quantity of betaxanthins (101.41 and 100.29 mg of pigment per 100 g of freeze-dried red beet). Additionally, the incorporation of ascorbic acid preserves the extracts from additional decomposition [36].

da Silva et al. [38] evaluated the optimal extraction conditions with UAE to isolate betalains in red beetroot (*B. vulgaris* L.). UAE is a non-thermal method that applies acoustic energy to enhance the liberation and diffusion rates of target materials by causing cavitation in the solvent. This method disrupts the solid matrix’s cell walls, enabling better solvent infiltration and mass transfer, which in turn enhances the extraction efficiency [38,45]. Compared to MAE, the primary advantage of UAE is that it operates at ambient temperatures, thereby preventing thermal overexposure [39]. The extraction was performed by placing 1 g of the dry sample into 25 mL of extraction solvent (water and ethanol mixture) in 250 mL Erlenmeyer flasks. An ultrasound indirect bath operating at 165 W and a frequency of 25 kHz, connected to a condenser, was utilized. Samples were subsequently strained, and the filtrate was promptly evaluated following extraction. The outcomes showed that the optimal extraction conditions for betacyanins and betaxanthins were acquired at temperatures of 52 °C and 37 °C, with a duration of 90 min, applying a solvent comprising 25% ethanol in water, which resulted in 4.2 mg g^−1^ of betacyanin and 2.80 mg g^−1^ of betaxanthin. The concentrations of betalains, phenolic compounds, and antioxidant activity obtained were superior to those achieved through conventional extraction methods, indicating that UAE is more efficient than extraction by conventional methods [38].

UAE was also applied by Laqui-Vilca et al. [46] for the extraction of betalains from colored quinoa (*Chenopodium quinoa Willd*) hulls. Colored quinoa hulls have been identified as a promising source of phenolics, flavonols, and betalains. It is essential to establish specific extraction conditions with UAE for each plant source, considering that the optimal conditions may vary depending on the composition of the matrix and the chemical characteristics of betalains. The UAE of betalains was conducted in tubes using a quinoa hull to water ratio of 1:100, and further optimization was performed utilizing response surface methodology (RSM), adopting a Box–Behnken design. The response surface plots illustrate the correlation between UAE variables such as amplitude, cycle, and extraction duration and the overall betalain composition. The optimal ultrasound treatments of amplitude = 70%; cycle = 0.6 (power is activated for 0.6 s followed by a pause of 0.4 s) and a brief extraction duration of 9.2 s yielded 96.477 mg of betacyanins/100 g fresh weight of sample (FW). Whereas the treatments of amplitude = 90%, cycle = 0.7 (power is activated for 0.7 s followed by a pause of 0.3 s), and short extraction time of 40 s rendered 201.01 mg of betaxanthins/100 g FW. Conventional extraction necessitated 30 min at ambient temperature to achieve comparable outputs [46].

A study indicated that β-cyclodextrin enhanced UAE facilitated the recovery of red beetroot extract, which can increase the extraction efficiency and maintain betalain stability for implementation in nutraceutical, food, and medical domains. β-cyclodextrin (β-CD) is a cyclic oligosaccharide derived from starch through enzymatic modification, comprising seven glucose units bonded by α-1,4 glycosidic bonds [47]. In aqueous solutions, β-CD serves as an extraction medium that enhances the efficient utilization of natural plant materials facilitated by non-covalent interactions with bioactive compounds. To extract the betalains, the processed red beet powders were combined with extraction solvents—water, aqueous ethanol solution (80% *v*/*v*), aqueous β-CD solutions (1 and 5% *w*/*v*) or ethanol solution of β-CD (1 and 5% *w*/*v*)—at a ratio of 1:10 *w*/*v* and mixed for three hours. The samples were added to an ultrasonic bath (28 kHz, 80 W, without applying external heat). The obtained extracts were centrifuged at a speed of 7000 rpm for ten minutes and filtered using Whatman No. 1 paper. Lastly, the extracts underwent lyophilization for the purpose of obtaining a dry extract. The red beet extracted with aqueous 5% β-CD solutions showed the greatest betanin quantity (2.243 ± 0.04 mg) [48].

Nutter et al. [49] also took advantage of the UAE for the retrieval of betalains from beet leaves. Beet leaves constitute approximately half of the entire plant and are typically disposed of as waste. This results in the loss of half of the biomass post-harvest and the wastage of various resources invested in production before reaching consumers. The extraction was carried out using water as the extraction solvent, under various conditions: ultrasound power (UP) ranging from 10 to 90 W, solid-to-liquid ratio values from 1:20 to 3:20, and extraction times from 4 to 16 min. The optimal conditions for extraction from beet leaves were 90 W UP, a 1:20 solid-to-liquid mixture, and an extraction duration of 16 min. These conditions substantially improved the recovery efficiency and reduced the extraction time compared with traditional maceration, resulting in 949.1 ± 28.4 µg g^−1^ betaxanthins and 562.2 ± 41.3 µg g^−1^ betacyanins [49].

A study reported the use of UAE with deep eutectic solvents (DES) for the obtention and preservation of betalains from beetroot wastes (peel and pulp). DES have recently been utilized as another solvent option for isolating biomolecules. DES are mixtures containing two or three elements, exhibiting characteristics akin to ionic liquids. These include lower melting points compared to their individual constituents, thermal stability, low vapor pressure, excellent ionic conductivity, adjustable viscosity, and compatibility with various solutes due to their miscibility and solubility properties. Furthermore, their modifiable polarity enhances the durability and selectiveness of extracts, as well as the extraction output [50]. DES were developed utilizing magnesium chloride hexahydrate [MgCl_2_·6H_2_O] as hydrogen bond donor and urea [U] as hydrogen bond acceptor to attain eutectic mixtures of [MgCl_2_·6H_2_O] [U] with suitable characteristics for extracting and preserving the betalains. 0.5 g of fresh beet pieces were combined with the DES at a solid-to-liquid ratio of 1:30 g/mL using a blender. Subsequently, UAE was carried out using an ultrasonic bath at 25 °C for three hours, followed by vortex stirring for 900 s. The liquid was then recovered from the beetroot material through the use of Whatman filter paper No. 1 for filtration. The study showed that extraction with DES solvent (2:1) and acidic pH produced the highest yield of betalains, specifically 296.7 ± 7.6 mg L^−1^ of betacyanin and 98.5 ± 7.5 mg L^−1^ of betaxanthin. Other authors have also verified the improvement of betalain extraction with acidification (pH 3–5) using water as the solvent. The betalains from DES extracts were well protected under visible light for 150 days and for 340 days during storage in amber vessels [50].

Tabio-García et al. [51] also employed UAE to obtain betalains from *Amaranthus hypochondriacus* var. Nutrisol is also recognized as a provider of betalains and various bioactive compounds. Amaranth powder was incorporated with distilled water at a 1:30 g/mL solute-solvent mixture, with the pH adjusted to 5 using 0.1 N HCl. Subsequently, the mixture underwent UAE at different temperatures and ultrasonic power densities (UPD) for 10 min. The combined conditions of temperature (41.80 °C), UPD (188.84 mW/mL) and extraction time (10 min) yielded 83.39% of total betalains, which was 1.38 times higher than conventional extraction. The findings indicate that UAE is a viable option, facilitating increased betalain yields in a more efficient period compared to standard methods [51].

Traditional extraction methods were conducted by Zin et al. [34] to obtain betalains from beetroot peel, aiming for a simple and cost-effective process, while also achieving a relatively high yield. They studied the impacts of process variables, including extraction time, temperature, and solvent ratio, on the quantities of betalains. They mixed 50 g of the beetroot pulp utilizing 15% aqueous ethanol in multiple peel-to-solvent configurations before carrying out the extraction procedures using a thermostat water bath to maintain temperatures of 20, 35, and 50 °C with three different durations (1, 3, and 5 h) and stirring at 215 rpm. It was obtained that the most suitable extraction conditions were extraction time of one hour, running temperature of 20 °C, and solvent concentration of 0.8 *w*/*v*, which yielded 1361 mg L^−1^ of betacyanin and 925.5 mg L^−1^ of betaxanthin. Lowering the extraction temperature and duration was favored to achieve improved yield. This preference can be attributed to the thermal sensitivity of natural color compounds [34].

Another technique that can be employed to obtain bioactive compounds from plants at near-room temperature is SFE. SFE is performed by employing supercritical fluids as the extracting solvent. Generally, a substance is referred to as a supercritical fluid when it is maintained at pressures and temperatures beyond its critical points. The molecular permeability coefficients and viscosity of supercritical fluids closely resemble those of gases, enabling them to flow easily like gases. Moreover, supercritical fluids exhibit very low surface tension, which enhances mass transfer intensity by improving the contact surface between phases in separation processes. This method significantly reduces sample preparation time and facilitates efficient recovery of analytes from liquid, solid, and semi-solid samples [44,52]. Carbon dioxide (CO_2_) is frequently used as a solvent in SFE due to its inert nature and safety (non-flammability, non-explosiveness), affordability, non-corrosiveness, lack of odor and color, and absence of residues in the final product. Its low surface tension, low viscosity, and high diffusivity further enhance its suitability as a solvent in SFE.

Moghimi et al. [52] obtained betalain from beetroot through SFE. Before the extraction procedures, the sample was preheated in a microwave oven for 90 s at 100–450 W. Subsequently, 10 g of the sample was extracted, with adjustments to the temperature (30–80 °C), pressure (15–40 MPa), and CO_2_ flow rate (1–3 mL/min). The optimal condition was achieved at a temperature of 45 °C, microwave power of 300 W, pressure of 27.5 MPa, and CO_2_ flow rate of 2 mL/min, resulting in 76% of total betalain. Additionally, the utilization of microwave-enhanced extraction efficiency facilitates cellular degradation. This shows that microwaves and supercritical fluids are effective extraction methods that retain heat-sensitive phytochemicals [52]. 

The isolation methods that have been used for the extraction of betalain from various sources are summarized in Table 1.

### 2.4. Application of Betalain as Colorimetric and Biosensor

Colorimetric analysis is a practical method utilized for quantifying the concentration of colored substances in a solution within the visible spectrum of light (400–800 mm). Colored substances absorb light within the visible spectrum, correlating directly with their concentration in the solution [53]. The concentration of the analyte can be determined with the use of a colorimeter/visible spectrophotometer, which measures the absorbance of a specific wavelength of light [54]. Visual evaluation may also be carried out using a score scale, which demands attention from the evaluator due to its difficulty [55]. The color of the sample is an inherent attribute of the solution, or it may be developed by the incorporation of appropriate reagents [54].

Colorimetric reagents are useful in determining many pharmaceutical substances in dosage forms and biological matrices with the help of spectrophotometric and chromatographic methods [56]. The basic principle of colorimetric reagents is that upon interaction with the target analyte, the change in color will provide information on the type of analyte as well as the concentration range. The coloration visualization may be accredited to the presence of different ligands from the reagent that form chelation with the analyte [57].

Biosensors are small analytical devices used to quantify biological or chemical reactions by creating signals to reflect the analyte concentration in a reaction. They are autonomous integrated devices capable of presenting qualitative and semi-quantitative analytical data using a biological recognition component that is combined with a transduction component. Biosensors are widely used for improving the quality of life, with wide-ranging applications such as environmental monitoring, disease diagnosis, drug discovery, and many more [58,59].

Biosensors usually consist of a biosensing element, a transducer, and a signal processing unit [59]. The biosensing element or bioreceptor is generally made up of an immobilized biocomponent capable of detecting the specific target analyte, mainly composed of microorganisms, nucleic acids, antibodies, enzymes, and cell receptors [59,60]. The transducer transforms the biological signals into optical and electrical signals correlated to the number of analyte-bioreceptor interactions. Based on its operational principle, the transducer can be classified as electrochemical, electronic, thermal, optical, and gravimetric transducers [59,61]. The signal processing unit amplifies the signal from the transducer so that the corresponding response can be processed and quantified [59].

## 3. Betalain as Colorimetric Sensor

Betalain can be colorimetric sensors due to their color change, which is influenced by their instability to pH less than 3 or higher than 7, high water activity, metal cations, high temperature, light, oxygen, hydrogen peroxide, and minimum pigment concentration [62]. Structurally, they remain stable between pH 3 and 7, and they have shown halochromic properties in alkaline conditions. The structure of betalain changes from anionic to cationic states, and its color will change from red to violet at pH levels less than 3, resulting in an apparent color shift from red to a blue-violet shade. When the pH is higher than 7, betalain’s aldimine bonds hydrolyze, producing betalamic acid and cyclo-dopa-5-O-glucoside, which cause the color change to yellow-brown [63]. 

### 3.1. Metal

Recent studies have reported that different types of metal detectors have been developed using betalain as colorimetric sensors as shown in Table 2.

Metal such as copper (Cu^2+^) can be found in aqueous-organic solutions such as methanol, acetonitrile, or acetone. These solvents are commonly used in studies on the pigments in analytical and organic chemistry. To determine the stability of 2-decarboxy-betanin, a range of degradation studies have been conducted, influenced by the effects of pH (3–8) in organic solvents and varying concentrations of copper ions. Skopińska et al. [12] investigated the impact of Cu^2+^ cations in ethanolic and methanolic solutions on 2-decarboxy-betanin, as one of the most degradative metal ions. The stability of 2-decarboxy-betanin is negatively impacted by an increase in Cu^2+^ cation concentration. The Cu^2+^ cations in organic solvents cause pigment degradation, whereas increasing the concentration of organic solvents promotes pigment decomposition. Spectra change and a significant decrease in absorbance are observed in molecules with absorption maxima at λmax approximately 430 nm, which are the primary degradation products of 2-decarboxy-betanin [12].

Metals are not only found in aqueous-organic mixtures, but also in water. A study reported by Cao et al. [64] betalain can be used as a colorimetric sensor to detect Cu^2+^ in drinking water using red beet pigment and a smartphone. Red beet pigment’s color transitioned from purple to orange-red in alkaline conditions due to a selective reaction between redox reaction and chelation with Cu^2+^. A smartphone was used to record the color change, and the application processed the image and presented Cu^2+^ concentration in the detection system. After analysis of the red beet pigment solution’s blue (B) value, it was discovered that a strong linear correlation existed between B value and Cu^2+^ concentration within the 4–20 μM range, with a detection limit of 0.84 μM. Furthermore, an Android-based smartphone application was created to visually detect Cu^2+^ by determining the colorimetric probe solution’s B value. The standard-addition method was utilized to successfully verify the practicality of this method in the identification of Cu^2+^ in drinking water [64].

Detecting metal in water was also reported by Hashemi et al. [65] who developed green practices for carbon dots (CDs) synthesis using natural precursors from red beetroot for detecting metal in water. CDs are a polymeric, semiconductor, and carbon nanomaterials used in biosensors, bioimaging, and biomedicine. Due to their minimal toxicity, temperature stability, biocompatibility, excellent quantum yield, solubility in water, and ease of synthesis from a broad range of carbon sources, red beetroot is used as a natural precursor to create fluorescent CDs that can be utilized to detect palladium ions (Pd^2+^) in mineral and well water. Through a static quenching process with a detection limit of 33 nM in a linear range from 3 μM to 43 μM, the addition of Pd2+ to the sample caused the CDs’ fluorescence to be quenched. Furthermore, all samples had acceptable recovery rates between 96.6 and 105.0%, with an RSD between 1.74 and 4.91%. These results imply that the suggested fluorescent probe can be a reliable and efficient option for determining the concentration of Pd^2+^ ions in water [65].

A similar study by Guo et al. [66] reported the synthesis of fascinating multifunctional carbon dots (CDs) with facile solvothermal treatment of red pitaya peels in acetic acid to prepare blue emission CDs (ACDs) for auric ion (Au^3+^) detection. When Au^3+^ was added, the as-prepared ACDs’ fluorescence intensity was rapidly, considerably, and selectively quenched, and the color of the solution shifted from yellow to purple with a maximum absorption at 545 nm. With detection limits of 0.072 μM and 2.2 μM, the fluorescence quenching ratio and absorbance show a linear change as Au^3+^ concentration increases within the ranges of 0.3–8.0 μM and 3.3–60.0 μM, respectively. The assay was used to determine Au^3+^ in spiked real water samples, and the results showed that fluorometric and colorimetric sensors were very accurate, highly precise, and incredibly selective for determining Au^3+^ in real samples such as river water and laboratory tap, with recoveries rates between 95.5 and 105% and a relative standard deviation (RSD) bellow 6.5% [66].

### 3.2. Nonmetal

These days, food product distribution and preservation depend significantly on packaging methods. This has created a new packaging system that can provide consumers with trustworthy information on food quality in a clear, concise, and easy-to-understand way. The ability to detect food freshness or the presence of gases in packing materials has been demonstrated by time-temperature-based color shifts [67]. Due to microbiological and chemical processes in food degradation which can release compounds such amines, ammonia, hydrogen sulfide, carbon dioxide, and organic acids, Naghdi et al. [68] reported pH and ammonia sensitivity in food packaging labels by detecting ammonia (NH_3_) through the addition of betacyanin from paperflower (Bougainvillea glabra) to potato starch film made using the solvent casting process. Based on FTIR analysis, it was shown that the betacyanin was evenly distributed in the starch matrix and had developed new reactions with it. When the pH was adjusted from 2 to 13, the film containing betacyanin changed from light pink to yellow. Additionally, ammonia in the range of 0.1 and 0.01 mg per milliliter of water could be detected by it. A visual change in the packaging label’s color from pink to yellow correlates with an elevation in total volatile basic nitrogen (TVB-N) [68,69]. The recent studies on nonmetal ion detection using betalains are summarized in Table 3.

A similar study by Yao et al. [69] reported active and smart packaging films were made by separately incorporating plant extracts containing high amounts of betacyanin (red pitaya flesh extract (RPFE), prickly pear fruit extract (PPFE), red beetroot extract (RBRE), globe amaranth flower extract (GAFE), and red amaranth leaf extract (RALE) to starch or polyvinyl alcohol. The shrimp’s freshness was shown by the packaging films through the detection of ammonia (NH_3_). Volatile ammonia and buffer solutions with a pH of 8–12 can affect all betacyanin-containing films. Interestingly, as shrimp spoiled, the RPFE-containing film showed noticeable color changes (from purple-red to pink) [69].

As well as a study conducted by Gao et al. [70], intelligent food packaging with κ-carrageenan-based pH-sensing films is used to provide real-time pork tenderloin product quality monitoring. Carrageenan is utilized as a non-toxic polymer matrix in food packaging to create edible films that prolong shelf life and improve food safety. The food packaging κ-carrageenan films were combined with anthocyanins, betacyanins that were derived from purple sweet potatoes, dragon fruit peels, and their mixture of dyes. The films’ ammonia sensitivity, oxidation stability, water vapor permeability, and UV protection capability were all significantly enhanced by betacyanins. It detected the indicator film’s color change and monitored the pork’s physical and chemical indexes while it was being stored. The release of volatile nitrogen compounds in meat during spoiling is shown by the color change in the betacyanin combination film from red to green, according to the results [70].

Recent studies have reported a new indicator film containing betacyanin (BC) from beetroot peel extract and carboxymethyl-cellulose (CMC)/flaxseed gum (FG) was conducted by Chaari et al. [71]. Unexpectedly, CMC/FG/BC film demonstrated a changing color transition from pink to yellow when the pH was adjusted from 1 to 12. The recently developed film showed remarkable sensitivity to ammonia solution concentrations in addition to pH sensitivity; when 0.5 mg/mL was added, it detected a variation from 0.125 to 0.5 mg/mL with a substantial ΔE (*p* < 0.05) decrease. On the other hand, after 14 days of storage at 4 °C, the CMC/FG/BC film’s potential to provide real-time monitoring of minced beef meat quality was tested. A significant color shift from light pink to purple was seen in the CMC/FG/BC film during the storage period, which correlated to changes in the total volatile basic nitrogen (TVB-N) of the beef samples [71]. 

## 4. Betalain as Biosensor

As mentioned before, a biosensor generally comprises a biosensing element combined with a transducer and a signal processing unit, and therefore operates on the basis of signal transduction [59]. Betalains can serve as biosensors, due to their fluorescence, which generates a robust and reliable signal easily detectable using conventional fluorescence equipment. Betaxanthins naturally fluoresce within the visible electromagnetic spectrum, rendering them an appropriate output signal for biosensors provided that the production of betalamic acid is engineered [16]. A range of biosensors involving the use of bacteria and yeast have been developed, due to their ability to convert intracellular product formation into a detectable optical signal. Betaxanthin is mostly used as the output of these cell-based biosensors due to considerations of practicability and cost-effectiveness [16,17,19,20].

### 4.1. Bacteria

Chen et al. [16] established a whole-cell biosensor involving the use of betaxanthin as output signals for monitoring environmental copper levels. The bacteria *Cupriavidus metallidurans* and *Ralstonia eutropha* were used to convert a copper-sensing signal into protein expression and emission of light facilitated by betaxanthins. These betaxanthins, exhibiting uniform optical properties, resulted from a spontaneous reaction between free amines and betalamic acid generated by DOPA dioxygenase (DOD). DOD is a plant enzyme present in Caryophyllales, responsible for converting L-DOPA into yellow, intensely fluorescent betaxanthins. The biosensor plasmid containing *pBAD-Mjdod* was transformed into the cells, allowing the generation of a yellow pigment noticeable to the human eye. The authors observed that betaxanthin could generate both fluorescent and colorimetric signals within a 6 h timeframe. This novel biosensor exhibited excellent performance in detecting Cu (II) in laboratory settings, including environmental samples such as pond and tap water [16]. The development of biosensor using betalains, involving bacteria are summarized in Table 4.

A similar approach was adopted by Lin and Yeh [17], by designing a whole-cell biosensor using the bacteria *Escherichia coli*, involving the use of betaxanthin as output signals for the analysis of dopamine (DA). DA, a principal catecholamine neurotransmitter, is known to be integral to various cognitive functions. Researchers have extensively studied the correlation between alterations in DA neurotransmission and neurodegenerative disorders, such as Parkinson’s and Huntington’s diseases. The biosensor was designed by using DA to initiate FeaR-regulating promoters in the bacteria, thereby inducing the production of red fluorescent protein (RFP), which can be detected by the naked eye. DA served as a substrate for DOPA 4,5-dioxygenase in order to produce 6-decarboxylated betaxanthin pigments, measurable by absorbance at 432 nm (A_432_). The proposed system exhibits a selective response to DA, with a minimum detection level of 11.1 µM. Subsequently, a dual-signal biosensor was designed, incorporating both RFP and Mjdod-His, and was regulated by the TynA promoter. This design allowed for the differentiation of DA from other common catecholamine neurotransmitters, including phenylethylamine (PEA), L-DOPA, and epinephrine (EPI). The dual output signals—RFP and betaxanthin pigment absorbance at 432 nm—enabled the distinction among catecholamines. PEA and L-DOPA each yielded either RFP or A_432_ signals as outputs, while DA generated both RFP and A_432_ signals, and EPI did not produce either [17].

For the detection of L-DOPA, which serves as a precursor of several catecholamine neurotransmitters, including DA, Chou and his group [18] developed an enzyme-based and a whole-cell-based biosensor. Plasmids containing DOPA 4,5-dioxygenase present in *Mirabilis jalapa* were transformed into *E. coli* BL21. For the enzyme-based method, IPTG was introduced to stimulate protein expression before the proteins were extracted and purified. Absorbance spectroscopy was utilized to evaluate enzyme activity, where the occurrence of a yellow color indicated the production of betalamic acid. The incorporation of L-DOPA led to a significant rise in absorbance at 430 nm, with a minimum detection level of 2.8 µM. Because color in betanin derivatives is regulated by structural changes in the cyclo-DOPA segment, betalamic acid was incubated with various primary amines. For the cell-based method, a detectable signal was visually obtained one hour following the incubation of the transformed cells with L-DOPA. In addition, the performance of the enzyme-based biosensor was validated in fetal bovine serum (FBS) as the experimental matrix. The authors assessed the enzyme’s efficacy in 33% serum samples spiked with L-DOPA. Comparable linearity, sensitivity, and dynamic range were detected in complex matrices, indicating the method’s potential applicability to biological specimens [18].

### 4.2. Yeast

A study reported by DeLoache et al. [19] demonstrated the development of an enzyme-coupled biosensor specific for L-DOPA by taking advantage of the production of betaxanthin in *Saccharomyces cerevisiae*. They expressed DOD from the plant *M. jalapa* along with the enzymes AbPPO2 from *Agaricus bisporus* and CYP76AD1 from *B. vulgaris*, which enabled the production of betaxanthin that could be readily detected visually. Culture supernatants from strains expressing both DOD and a CYP76AD1 mutant were collected following growth in minimal medium supplemented with ascorbic acid to prevent betanidin oxidation. The sensor exhibited a broad dynamic range of sensitivity, covering 110-fold from its lowest to highest detectable signals. Moreover, it demonstrated consistent performance across various samples, achieving a Z’-factor of 0.91. A Z’-factor within the range of 0.5 to 1.0 typically signifies an assay’s suitability for effective high-throughput testing [19]. The development of biosensor based on betalain and yeast are summarized in Table 5.

A comparable approach was employed by Mao et al. [20] by devising a biosensor as a high-throughput method for identifying mutants with a desired phenotype. Genes responsible for tyrosine hydroxylation and DOPA dioxygenase were inserted into the wild-type *S. cerevisiae* BY4741 strain, resulting in the NK-WT1 strain, which facilitates the alteration of L-tyrosine into betaxanthin. Betaxanthin was employed due to its detectability via visual assessment and fluorescence analysis, facilitating the rapid identification of high L-tyrosine producers following random mutagenesis. An increased extracellular concentration of L-tyrosine produced a more intense coloration of the medium observed via fluorescence spectroscopy in the biosensor cells [20].

To allow analysis outside of a laboratory setting, Miettinen et al. [21] developed a novel betalain-based colorimetric reporter for portable biosensors, using *S. cerevisiae*. The three betalain-based (betanin, betaxanthin, and O-dianisidine-betacyanin) transcriptional reporters were constructed based on three different biosynthetic pathways. The DOPA dioxygenase from *M. jalapa* (*Mj*DOD) performed as the reporter gene regulated by the biosensor. The O-da-betacyanin showed the most distinguishable color that was detectable by eye and in absorbance at 520 nm. This sensor is capable of detecting any structurally diverse cannabinoids that function as cannabinoid receptor CB2 ligands, generating a measurable signal within 15 min. These portable biosensors are anticipated to be further developed into commercial devices through the optimization of cell phone accessory devices and software [21].

Another biosensor designed for on-site detection was reported by Fan et al. They utilized a yeast biosensor that minimized noise and enhanced signal readout for detecting copper ions. They reprogrammed the yeast *S. cerevisiae* to reduce the background interference often encountered in CUP1-mediated sensing systems in yeast with the aid of CRISPR/Cas9-mediated genome editing. Similarly to the previous studies, heterologous expression of CYP76AD1 from *B. vulgaris* and L-DOPA dioxygenase from *M. jalapa* was used to produce distinctive orange coloration upon exposure of yeast cells to Cu(II) concentrations exceeding 5 µM (~0.32 ppm). The visual effect could be notably intensified with the addition of 0.5 mM amino donors such as o-dianisidine (oDA). However, future work will be required before this yeast biosensor can be applied in practical settings due to the possibly interfering factors affecting the performance of the biosensor [22].

## 5. Conclusions

Betalain is a natural pigment categorized into two groups: betacyanins, which form red-violet pigments, and betaxanthins, which form yellow-orange pigments. The distinguishable color of betacyanins is efficiently utilized as colorimetric sensors. Betalain colorimetric sensors are widely used to detect metal compounds in water and nonmetals compounds in food. Metal compounds detection can be sophisticated, fast, and efficient by using smartphones; additionally, detection can be more sensitive and selective by using carbon dots. Likewise, the detection of nonmetallic compounds in food can monitor food freshness during its transportation and storage by interactions with the food and its direct environment. Betaxanthins are known to display natural fluorescence within the visible electromagnetic spectrum, which is utilized as an output signal in biosensors. This property holds promise for the development of betalain-based biosensors with numerous potential applications across environmental monitoring, healthcare, and biotechnology. Furthermore, integrating betalain-based sensors with digital technologies such as smartphone apps could enhance data analysis and enable real-time monitoring based on the sensor data. On the other hand, the fragile stability of betalains is a major challenge throughout the extraction, handling, and containment of these compounds. Therefore, future research could focus on incorporating innovative technologies as well as optimizing extraction parameters to achieve effective extraction. Additionally, research should explore not just the processes, but also the stability, bioavailability, bioactivity, and potential interactions of betalain with other compounds to improve the analytical capabilities of betalain-derived sensors, such as research into the application of betalain as novel bio-indicators or reagents for specific analytes could be promising.

## Figures and Tables

**Table 1:** Different extraction methods for betalain.

Source of Betalain	Extraction Method	Extraction Solvent	Extraction Conditions	Reference
Red beetroot (*B. vulgaris*)	MAE	Mixture of ethanol and water (1:1)	0.1 to 25 ratio of solids to solvent, power 400 W and 100% duty cycle for 90–120 s (betanins) and 140–150 s (betaxanthins)	[36]
Red beetroot (*B. vulgaris*)	UAE	25% of ethanol in water	Temperatures of 52 °C and 37 °C, extraction duration of 90 min	[38]
Colored quinoa (*Chenopodium quinoa* Willd) hulls	UAE	Water	Amplitude at 70%, cycle at 0.6, and extraction duration of 9.2 s for betacyanin and amplitude at 90%, cycle at 0.7, and extraction duration of 40 s for betaxanthin	[39]
Red beetroot (*B. vulgaris*)	UAE with β-cyclodextrin	Water, aqueous 5% β-cyclodextrin solution	Red beet powders mixed with extraction solvent (aqueous 5% β-cyclodextrin solution) at a 1:10 *w*/*v* ratio, extraction duration of 3 h, ultrasonic 28 kHz, 80 W	[48]
Beetroot (*B. vulgaris* L. var. conditiva) leaves	UAE	Water	Ultrasonic power of 90 W, 1:20 solid-to-liquid ratio, and extraction duration of 16 min	[49]
Beetroot (*B. vulgaris* L., Rhonda type) peels	Traditional extraction	Aqueous ethanol	Temperature of 20 °C, 0.8 *w*/*v* solvent mixture, and extraction duration of 1 h	[34]
Beetroot (*B. vulgaris*) waste	UAE using DES	Mixture of magnesium chloride hexahydrate [MgCl_2_·6H_2_O] and urea [U] (2:1) and pH 3	Temperature of 25 °C for 3 h, followed by subsequent vortex agitation for 900 s	[50]
*Amaranthus hypochondriacus* var. Nutrisol.	UAE	Distilled water	Temperature 41.80 °C and UPD 188.84 mW/mL for 10 min	[51]
Beetroot (*B. vulgaris*)	SFE	Carbon dioxide	Temperature 45 °C, microwave power 300 W, pressure 27.5 MPa, and CO_2_ flow rate 2 mL/min	[52]

**Table 2:** Studies on metal ion detection by betalains.

Pigment	Analyte	Sample	Color Change	Detection	Parameter Performance	Reference
2-decarboxy-betanin pigment	Cu^2+^	Aqueous-organic solutions (EtOH and MeOH)	Decreased pigment retention	UV-Vis Spectrophotometer	Not determined	[12]
Red beet extract	Cu^2+^	Drinking water	Purple to orange-red	Naked eye	Linearity range of 4–20 μM, with a detection limit of 0.84 μM	[64]
Red beetroot	Pd^2+^	Well water and mineral water	Quenched green-emitting CDs fluorescence	Fluorescent	Limit of detection of 33 nM in a linear range from 3 μM to 43 μM	[65]
Red pitaya peel (*Hylocereus polyrhizus*) extract	Au^3+^	River water and laboratory tap	Quenched blue-emitting CDs fluorescence and yellow to purple	Fluorescent and naked eye	Limit of detection of 0.072 μM at 0.3–8.0 μM Au^3+^ and 2.2 μM at 3.3–60.0 μM.	[66]

**Table 3:** Studies on nonmetal ion detection by betalains.

Pigment	Analyte	Sample	Color Change	Detection	Parameter Performance	Reference
Paperflower (*Bougainvillea glabra*) extract	NH_3_	Caspian sprat	Light pink to yellow	Naked eye	Liniearity range of 0.1–0.001 mg/ml	[68]
Red pitaya (*H. polyrhizus*) extract, prickly pear fruit extract, red beetroot (*B. vulgaris* L.) extract, globe amaranth flower extract, red amaranth (*A. tricolor* L.) extract	NH_3_	Shrimp	Purple-red to yellow on red pitaya extract	Naked eye	Response time of SP-GAFE film is 20 min	[69]
Purple sweet potato and Dragon fruit peel extract	NH_3_	Pork tenderloin	Red to green	Naked eye	Response time of 15 min	[70]
Beetroot peel (*Beta vulgaris* L.) extract	NH_3_	Raw beef meat	Pink to yellow	Naked eye	Response time of 30 min	[71]

**Table 4:** Development of biosensor involving betalain and bacteria.

Analyte Sensed	Type of Sensor	Property of Betalain Exploited	Performance Parameters	Reference
Environmental copper (freshwater and tap water)	Whole-cell based	Betaxanthin fluorescence	Response time of 6 h and linearity range at 0–250 µM	[16]
Dopamine	Whole-cell based	Betaxanthin fluorescence	Detection limit of 11.1 µM and linearity range at 0–2000 µM	[17]
L-DOPA in physiological fluids	Enzyme-based and whole-cell-based	Betaxanthin fluorescence	Detection limit of 2.8 µM (enzyme-based), response time of 1 h (whole-cell based), and linearity range at 5–500 µM	[18]

**Table 5:** Development of biosensor involving betalain and yeast.

Analyte Sensed	Type of Sensor	Property of Betalain Exploited	Performance Parameters	Reference
L-DOPA	Enzyme-coupled	Betaxanthin fluorescence	Linearity range at 2.5–2500 µM	[19]
L-tyrosine high-yield mutant cells	Whole-cell based	Betaxanthin fluorescence	-	[20]
Cannabinoids	Whole-cell based	Betanin, betaxanthin, and betacyanin fluorescence	Detection limit of 100 pM and response time of 15 min	[21]
Environmental copper	Whole-cell based	Betaxanthin fluorescence	Detection limit of 0.32 ppm	[22]

## Data Availability

All data are contained within the article.

## Supplementary Materials

The following supporting information can be downloaded at: https://www.mdpi.com/article/10.3390/bios15060349/s1, Table S1: Chemical structure of betalain types.
