# Supplementary material for: Betalain Pigments: Isolation and Application as Reagents for Colorimetric Methods and Biosensors
Source: Biosensors (Basel). 2025 Jun 1;15(6):349. doi: 10.3390/bios15060349 (PMC12190680; doi:10.3390/bios15060349)
Supplement: Supplementary file 1 [file biosensors-15-00349-s001.zip › biosensors-3631483-supplementary.pdf]

Review

Betalain Pigments: Isolation and Application as Reagents for Colorimetric Methods and Biosensors

Rimadani Pratiwi *, Devita Salsa Maharani and Sarah Gustia Redjeki

Department of Pharmaceutical Analysis and Medicinal Chemistry, Faculty of Pharmacy, Universitas Padjadjaran, Sumedang 45363, Indonesia

* Correspondence: rimadani.pratiwi@unpad.ac.id

Table S1. Chemical structure of betalain types

Types of Betalains	Chemical Structure
Betaxanthin	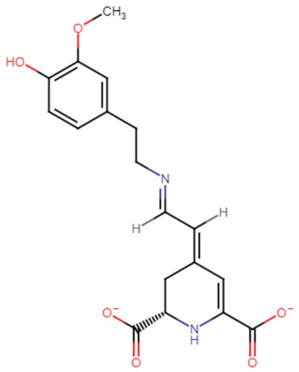
Indicaxanthin	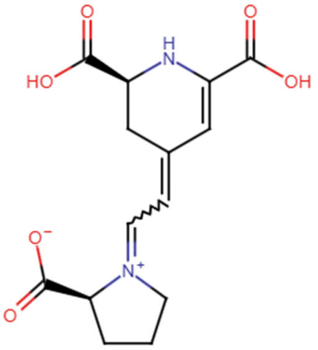
Miraxanthin I	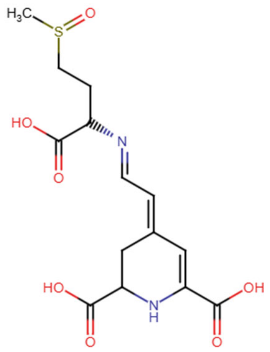

Miraxanthin II	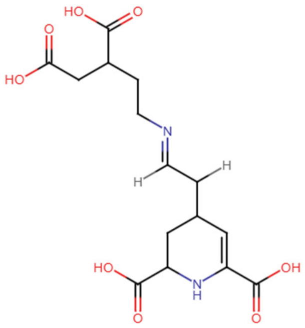
Miraxanthin III	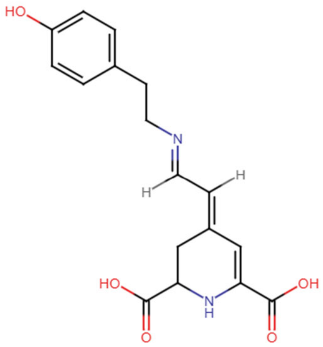
Miraxanthin V	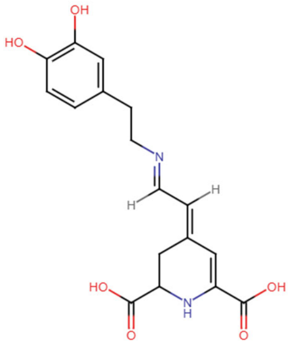
Vulgaxanthin I	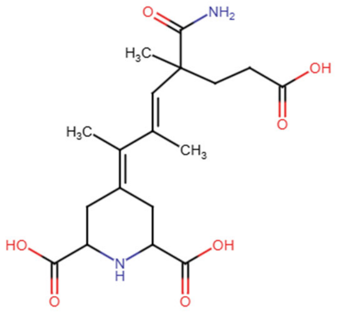

Vulgaxanthin II	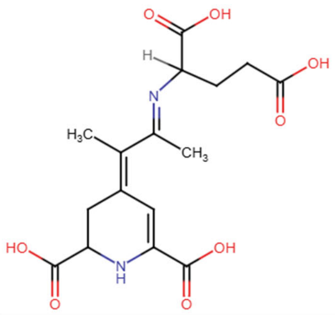
Portulacaxanthin I	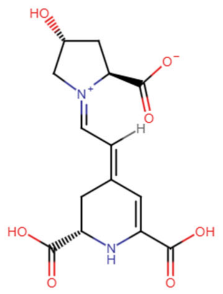
Portulacaxanthin II	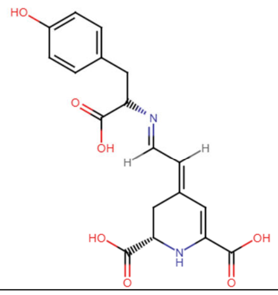
Portulacaxanthin III	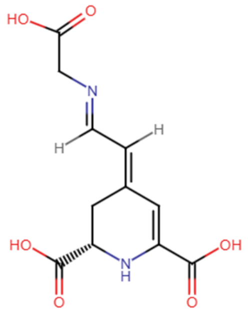
Betacyanin	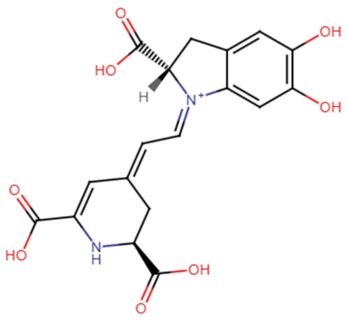

Gomphrenin I	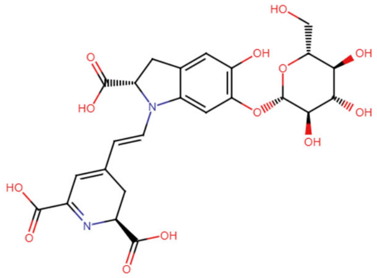
Gomphrenin II	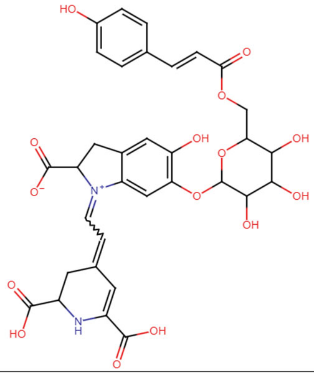
Gomphrenin III	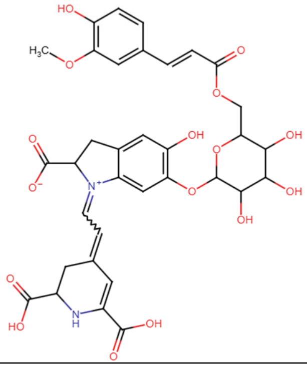
Gomphrenin V	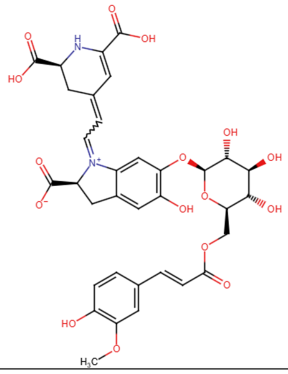

Bougainvillein	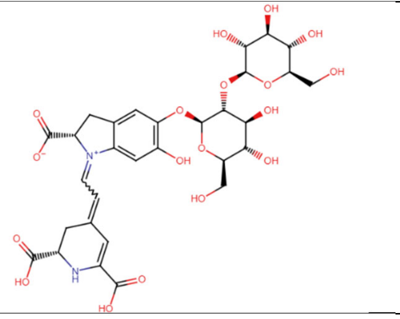
Celosianin	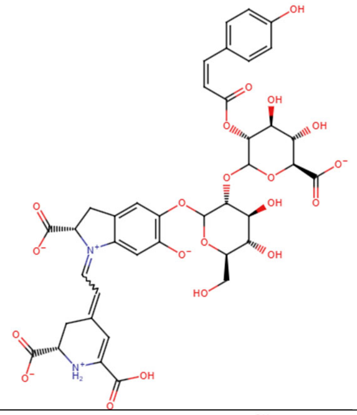
Iresinin	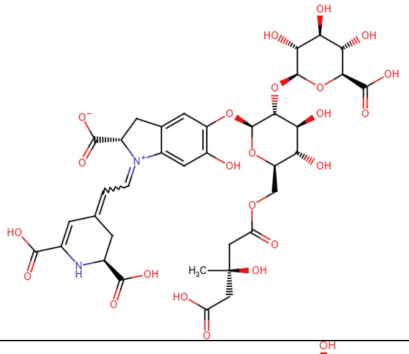
Amaranthin	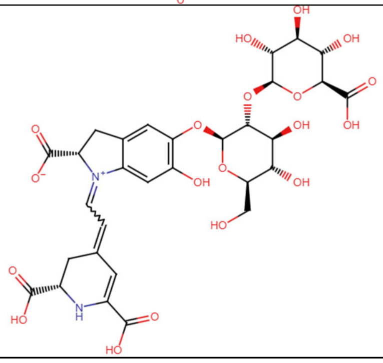

Neobetanin	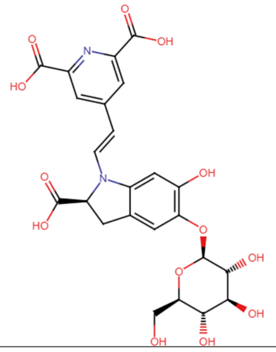
Betanin	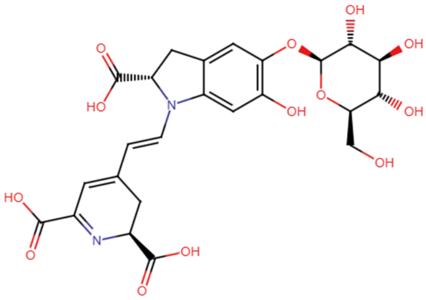
Prebetanin	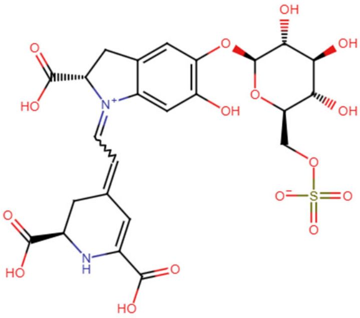
